# Complete Genome Sequences of Mycobacteriophages Apex and Gophee

**DOI:** 10.1128/MRA.01094-20

**Published:** 2020-11-05

**Authors:** Denzel Edwards, Tara Carney, Yunia Alvarez, Justin Pagan, Emma Sarro, Regina Alvarez, Bernadette J. Connors

**Affiliations:** a Department of Science, Dominican College of Blauvelt, Orangeburg, New York, USA; Queens College

## Abstract

Apex and Gophee are mycobacteriophages directly isolated from soil using the host Mycobacterium smegmatis mc^2^155. Apex has a 71,244-bp double-stranded DNA (dsDNA) genome encoding 98 putative proteins, and Gophee has a 68,556-bp dsDNA genome encoding 101 putative proteins.

## ANNOUNCEMENT

Phages Apex and Gophee were isolated directly from soil using Mycobacterium smegmatis mc^2^155 as the host. M. smegmatis is a widely used actinobacterium in the SEA-PHAGES program (seaphages.org) and has been used to isolate 1,946 phages (1). Isolation, purification, and amplification of these phages were done at 37°C using the double-layer agar method with Luria medium containing 1 mM CaCl_2_, 10 μg/ml cycloheximide, and 50 μg/ml carbenicillin. Plaques produced as a result of phage infection were not turbid. DNA was purified from a high-titer lysate (>5 × 10^10^ PFU/ml) using the Promega Wizard DNA cleanup kit. Agarose gel electrophoresis was used to verify the quality of extracted DNA. Sequencing libraries were prepared using the New England Biolabs (NEB) Ultra II FS kit with dual-indexed barcoding and sequenced using the Illumina MiSeq platform v3. For each genome, *de novo* assembly using Newbler v2.9 (2) produced a single circularized contig. These were checked for completeness, accuracy, and genomic termini using Consed v29 (3) as previously described (4). Approximate shotgun coverages for single-end 150-bp reads are shown in Table 1, along with other genome characteristics. Genomes were annotated using DNA Master (http://cobamide2.bio.pitt.edu/computer.htm), Glimmer (5), and GeneMark (6). Functions and functional synteny were predicted using BLASTp on the NCBI nonredundant (NCBI-nr) and Actinobacteriophage databases (E value of 10e-07 or less) and HHpred (probability value, >90%) (7–9). ARAGORN and tRNAscan were used to detect tRNA (10, 11), and transmembrane domains were detected using TMHMM v2.0 (12).

**TABLE 1:** Mycobacteriophage genome details and assembly results

Phage	GenBank accession no.	SRA accession no.	Length (bp)	G+C content (%)	No. of CDS^*a*^	Coverage (×)/no. of library reads	Database reference
Apex	MN428050	SRX9125695	71,244	68.9	98	3,561/1,785,118	phagesdb.org/phages/Apex/
Gophee	MH651175	SRX9125696	68,856	66.5	101	2,248/1,092,306	phagesdb.org/phages/Gophee/

a CDS, coding DNA sequences.

Phages that exhibit >50% nucleotide similarity are arranged into clusters and subclusters (13). Mycobacteriophages Apex and Gophee were placed in subclusters B4 (*n* = 15) and B1 (*n* = 198), respectively (*n* refers to the number of phages in the data set). Transmission electron microscopy (TEM) revealed siphoviral morphology with isometric heads. Isolation details and plaque and TEM images are available at phagesdb.org. Plaque and particle characteristics are expected of lytic phages in these subclusters (14).

Only 28% of genes in Apex and 26% in Gophee have annotated functions. The iconic synteny of the major tail protein, tail assembly chaperone, tape measure protein, and minor tail proteins is found in the first half of each genome (15). Their lysis cassettes consist of two lysins (A and B) but lack putative holins. Holins are reported to accumulate in the host cell membrane until such time that the membrane is permeabilized and endolysins then have access to the cell wall, resulting in the release of newly formed virions (16). Holin genes are ∼400 bp long, are positioned near lysins A and B, and encode a single transmembrane helix in most mycobacteriophage genomes. In subcluster B1, 5.1% of annotated genomes have putative holins, while 0% of those in the B4 subcluster have this feature. This is in significant contrast to two randomly chosen subclusters, F1 (*n* = 152) and C1 (*n* = 128), in which 77.6% and 43.8% of genomes, respectively, have putative holins. Interestingly, the majority of phages in subcluster B2 encode putative holins but not lysin B (73.1%, *n* = 26). Continued exploration of the lysis cassette in the B cluster may elucidate the evolution of genes responsible for virion release.

### Data availability.

The genome sequences reported here have been deposited in GenBank under the accession numbers listed in Table 1 (BioProject number PRJNA488469). The SRA numbers are also provided in Table 1.
